# Potential effects of intrinsic heart pacemaker cell mechanisms on dysrhythmic cardiac action potential firing

**DOI:** 10.3389/fphys.2015.00047

**Published:** 2015-02-23

**Authors:** Yael Yaniv, Kenta Tsutsui, Edward G. Lakatta

**Affiliations:** ^1^Biomedical Engineering Faculty, Technion-Israel Institute of TechnologyHaifa, Israel; ^2^Laboratory of Cardiovascular Science, Biomedical Research Center, Intramural Research Program, National Institute on Aging, National Institutes of HealthBaltimore, MD, USA

**Keywords:** arrhythmias, atrial fibrillation, coupled-clock pacemaker system, heart rate variability, sinus node disease

## Abstract

The heart's regular electrical activity is initiated by specialized cardiac pacemaker cells residing in the sinoatrial node. The rate and rhythm of spontaneous action potential firing of sinoatrial node cells are regulated by stochastic mechanisms that determine the level of coupling of chemical to electrical clocks within cardiac pacemaker cells. This coupled-clock system is modulated by autonomic signaling from the brain via neurotransmitter release from the vagus and sympathetic nerves. Abnormalities in brain-heart clock connections or in any molecular clock activity within pacemaker cells lead to abnormalities in the beating rate and rhythm of the pacemaker tissue that initiates the cardiac impulse. Dysfunction of pacemaker tissue can lead to tachy-brady heart rate alternation or exit block that leads to long atrial pauses and increases susceptibility to other cardiac arrhythmia. Here we review evidence for the idea that disturbances in the intrinsic components of pacemaker cells may be implemented in arrhythmia induction in the heart.

## Introduction

Normal cardiac impulse initiation and conduction are generated by specialized, self-excitable, pacemaker cells residing in the sinoatrial node (SAN). Defects in these cell-intrinsic capacities to elicit spontaneous action potentials (APs) can lead to disturbances of the rate and rhythm of heart beats, and can induce numerous clinical arrhythmia syndromes: (i) SAN dysfunction has been postulated to be a source of sinus nodal re-entry (i.e., reciprocal beats between SAN and atrium) tachyarrhythmia (Birchfield et al., 1957) which accounts for 2–17% of all arrhythmias (Cossu and Steinberg, 1998). While diagnosis of this arrhythmia is difficult, due to electrocardiographic similarity of the P-wave to the normal sinus rhythm (Gomes et al., 1995), microelectrode studies in isolated rabbit hearts (Han et al., 1968) and later in humans (Childers et al., 1973) indeed demonstrate that SAN is the source that induces re-entry. (ii) Sick sinus syndrome, characterized by symptomatic dysfunction of the SAN (reviewed in Dobrzynski et al., 2007), can be manifested as sinus bradycardia, sinus arrest, or SAN block, and in some cases supraventricular tachyarrhythmias (“tachy-brady” syndrome), atrial flutter or atrial fibrillation. In mice with an inducible phenotype that mimics sick sinus syndrome, heart beating intervals (BIs) were completely irregular both *in vivo* and in the isolated Langendroff perfused model (no brain-heart signaling) (Herrmann et al., 2007). Telemetric ECG recordings revealed a variety of arrhythmias: sino-atrial arrhythmia, sino-atrial pause and supraventricular or ventricular tachycardia. (iii) It has been suggested that abnormal stretch of the rat atria that accompanies many heart diseases (De Jong et al., 2013) and occurs even in transplanted human hearts (Slovut et al., 1998) (no brain-heart connection) induces respiratory sinus arrhythmia.

Here we review evidence for the idea that changes in the membrane and sarcoplasmic reticulum (SR) components of pacemaker cells may be implicated in arrhythmia induction in the heart.

## Intrinsic coupled-clock mechanisms to pacemaker cells control the heart rate and rhythm

To understand abnormal SAN function it is essential to first understand the normal function of intrinsic properties of pacemaker cells and their modulation by brain-heart signaling. Experimental and theoretical data over the past two decades indicate that pacemaker cells residing in the SAN entrain their AP BI variability (BIV) by regulation of intracellular electric and mechanical coupling (reviewed in Yaniv et al., 2013a).

The coupled-clock system (Yaniv et al., 2013b; Maltsev et al., 2014) that controls the pacemaker cell beating rate and rhythm consists of an intracellular “Ca^2+^ clock” and “M clock.” The sarcoplasmic reticulum Ca^2+^ pump and ryanodine channels act as a “Ca^2+^ clock,” discharging local Ca^2+^ releases (LCRs) close to the cell surface membrane; LCRs activate membrane electrogenic clock molecules (“M clock”), mainly the Na^+^/Ca^2+^ exchanger. Na^+^-Ca^2+^ exchange current, the f-channel current, and K^+^ channel current, other components of the M clock, concurrently drive the diastolic membrane depolarization to ignite the next AP. The Ca^2+^ and M clocks entrain each other through electrical and chemical signaling: Ca^2+^ activation of calmodulin -adenylyl cyclase (AC)-dependent protein kinase A (PKA) and Ca^2+^/calmodulin-dependent protein kinase II (CaMKII). Both of these signaling pathways affect phosphorylation of proteins of both clocks [i.e., phospholamban (PLB) and ryanodine receptors (Ca^2+^ clock) and L type and K^+^ channels (M clock)]. Additionally, cAMP positively shifts the f-channel activation curve. Based on the coupled-clock theory, a change in the activity or in the quantity of every molecule within the M or the Ca^2+^ clock or a change in the chemical coupling signaling of both clocks will perturb the function of the other clock, and thus alter the degree of entrainment between them. For example, in rabbit pacemaker cells, reduction in I_*f*_activity or in Ca^2+^ clock proteins leads to reduction in the coupled-clock phosphorylation activity and LCR signal (Yaniv et al., 2014b). Thus, a reduction of internal signaling within one clock can lead to a reduction in the degree of clock coupling and changes in the function of the other clock that change automaticity.

Even under normal conditions, spontaneous AP BIs of mammals including human pacemaker cells are not constant, but vary around the average AP BI, due to stochastic properties of intrinsic mechanisms of the coupled-clock system (Verheijck et al., 1998; Rocchetti et al., 2000; Zaza and Lombardi, 2001; Monfredi et al., 2013; Papaioannou et al., 2013; Yaniv et al., 2014b). The degree of AP BIV is related to variability in both the timing of LCR occurrence during the diastolic depolarization, and to the ensemble LCR Ca^2+^ signal: an increase in LCR variability is associated with a reduced ensemble LCR Ca^2+^ signal that occurs later in diastole (i.e., prolonging the next AP ignition). Based on the coupled-clock theory, the stochasticity of LCR periods (i.e., the times of LCR occurrences following the prior AP) not only depends upon stochasticity of spontaneous RyR activation, but also upon stochastic sarcolemmal ion channel openings and closings that regulate the cell Ca^2+^ balance. The amplitude and timing of LCR Ca^2+^ signals to M clock proteins report the efficiency of clock coupling, i.e., a weaker LCR signal to M clock proteins that occurs later in time reports less-efficient clock coupling. Consequently, changes in the steady-state AP BI and the BIV embody contributions of both clocks. Reduction in the degree of synchronization between the clocks disturbs the ability to maintain the basal average AP BI, leading not only to a reduction in the average AP BI, but also to an increase in variability around the prolonged average AP BI.

**Figure 1:**
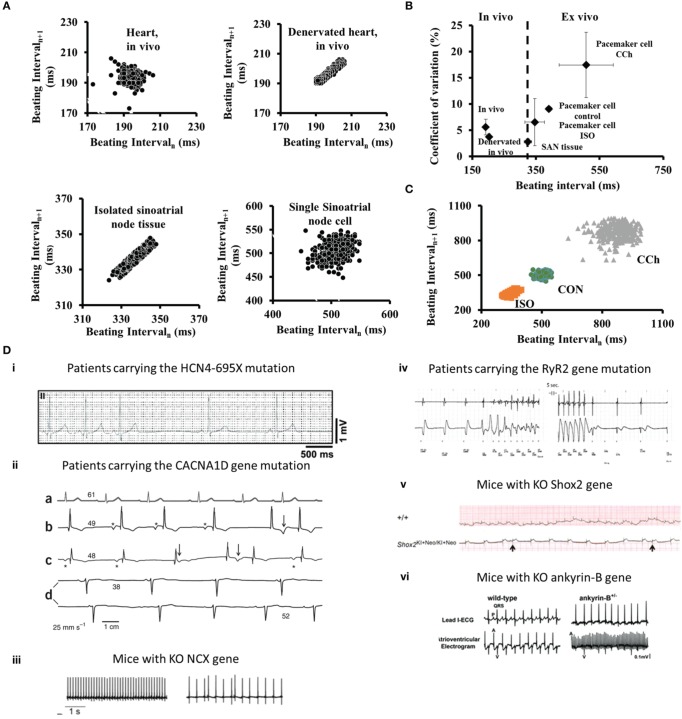
**(A) Poincaré plots of the beating interval at different levels of integration from the heart *in vivo* to single isolated pacemaker cells. (B)** The relationship between beating intervals and coefficients of variation at different levels of integration from the heart *in vivo* to single pacemaker cells in isolation. **(C)** Poincaré plots of the beating interval variability in single SANC under control (CON), β-adrenergic receptor stimulation (ISO) or cholinergic receptor stimulation (CCh). Modified from Yaniv et al. (2014a). **(D)** Examples of arrhythmias associated with changes in intrinsic clock mechanisms recorded *in vivo*: (i) in patients carrying the HCN4-695X mutation (adapted from Schweizer et al., 2010); (ii) (a–d) ECG recordings from an unaffected person (a) and three individuals who are homozygous for the CACNA1D mutation (b–d) (adapted from Baig et al., 2011); (iii) Cardiac arrhythmias in freely moving NCX KO mice (adapted from Herrmann et al., 2013); (iv) A rapid and presumably polymorphic ventricular tachycardia in patents with mutation in RyR2 (adapted from Bhuiyan et al., 2007); (v) ECG recording demonstrate the occurrence of arrhythmia in Shox2 KO mice (adapted from Liu et al., 2011); (vi) ECG recording from ankyrin-B KO mice (adapted from Cunha et al., 2011).

Autonomic neural input can entrain the rate and rhythm of electrical impulses that are generated by SAN tissue of mammals (Difrancesco, 1993; Boyett et al., 2000; Monfredi et al., 2014; Yaniv et al., 2014a). The balance between sympathetic to parasympathetic stimulation has a role in synchronizing intrinsic clock periods of individual pacemaker cells. β adrenergic receptor stimulation increases synchronization of intrinsic clock mechanisms leading to a decrease of both BI and BIV of pacemaker cells (Figures 1B,C). Moreover, β adrenergic receptor stimulation of a single pacemaker cell increases the probability that the beating intervals exhibit fractal-like behavior. Cholinergic receptor stimulation of pacemaker cells, on the other hand, decreases synchronization of intrinsic clock mechanisms, leading to an increase of both the average BI and BIV (Figures 1B,C).

## Synchronization of activity across the population of cells can impact on the heart rate and rhythm

Although we have focused here upon synchronization of mechanisms intrinsic to pacemaker cells, cell-to-cell interactions (electrotonic and mechanical) of pacemaker cells residing in SAN tissue also entrain the rate and rhythm of electrical impulses that emanate from the SAN (Jalife, 1984; Watanabe et al., 1995). These interactions have a role in synchronizing the intrinsic clock periods of individual cells (Sheikh et al., 2013), because the average range of basal AP BI and AP BIV of single isolated pacemaker cells is well above their range when they reside in rabbit SAN tissue (Yaniv et al., 2014a) (Figure 1A). When pacemaker cells are embedded within SAN tissue, those with the shortest AP BI create a primary pacemaker area within the SAN, leading to the origin of an electrotonic force that spreads to other SAN cells, resulting in the emanation of an electric impulse that excites the rest of the heart (Anumonwo et al., 1991). This impulse controls the heart rate and rhythm. When rabbit pacemaker cells are isolated from SAN tissue, their beating interval entropy increases dramatically compared to that when these cells reside in SAN tissue (Figure 1B), and fractal-like behavior of AP BIs, a feature that characterizes AP BI of SAN tissue, is absent in isolated single pacemaker cells (Yaniv et al., 2014a).

## Changes in heart rate variability indexes and the presence of arrhythmia

An increase in pacemaker cells AP BIV, or in mathematical terms coupled-clock-system entropy, above a certain threshold leads to abnormal impulse generation by the SAN that is defined as arrhythmia. Two regimes of heart rate variability (HRV) are analyzed in patients with arrhythmogenic events: during the events when the entropy of the system increases (Costa et al., 2008), and before the occurrence of arrhythmia. The occurrence of major arrhythmic events in patients with right ventricular cardiomyopathy is associated with a reduced BIV (Battipaglia et al., 2012). Interestingly, heart rate variability indexes decrease just prior to an arrhythmogenic event (Postolache et al., 2011). As we summarized here, the degree of synchronization of intrinsic mechanisms to pacemaker cells and the degree of synchronization among pacemaker cells within the SAN are determinants of the heart rate and rhythm. Autonomic receptors on pacemaker cells respond to the imbalances of autonomic impulses associated with cardiac diseases. Specifically, autonomic receptor stimulation of single pacemaker cells alters their beat-to-beat variability. Thus, intrinsic pacemaker mechanisms may contribute to HRV *in vivo*. Although mechanisms of HRV may vary from one patient to another, documentation of the relationship between HRV and different arrhythmias in human patients (Table 1) is an important initial step to conceptually link intrinsic pacemaker mechanisms to arrhythmogenic events. Note, that this sort of evidence, however, does not prove that altered synchronization of pacemaker clock mechanisms residing within the SAN are the sole cause of all patient arrhythmias that may be linked to changes in HRV.

**Table 1:** **Summary of primary studies assessing the changes of heart rate variability when arrhythmia occurs**.

**Type of arrhythmia**	**Number of patients**	**Findings**	**Study**
AF	27	Decreased pNN50 was an independent predictor of AF relapse	Akyurek et al., 2003
AF	784	Impaired LF spectral component predicted new-onset AF	Perkiomaki et al., 2014
AF	83	Time and frequency indices attenuated when the treatments failed	Seaborn et al., 2014
PSVT	64	HR increased, HRV and HF power decreased after catheter ablation for PSVT	Kocovic et al., 1993
Sick sinus syndrome	30	Poincare plot often showed random-like pattern;	Bergfeldt and Haga, 2003
		Beta coefficient^*^ of fractal increased toward 0 in sinoatrial node dysfunction	
Sick sinus syndrome	181	Decreased SDNN and rMSSD after pulmonary vein isolation	Wang et al., 2013
VF	24	HRV indices consistently did not change before VF	Vybiral et al., 1993
VF	15	VF patients had lower DFA alpha (0.64 vs. 1.05) and fractal beta coefficient^*^ (−1.63 vs. −1.31) than control	Makikallio et al., 1999
VT	40	All power spectra of HRV decreased before the onset of sustained VT compared to before nonsustained VT	Huikuri et al., 1993
VF/AVB	25	V-shaped trough appeared in the curve of ln(LF/HF) and ln(HF) prior to VF and AVB, respectively	Osaka et al., 2010
VF/AVB	292	Beta coefficient^*^ < −1.5 was the most powerful predictor of VF	Gang et al., 2011
VT/VF	312 after myocardial infraction	Decreased SDNN, VLF, HF, DFA alpha1 predicted VT/VF	Huikuri et al., 2009
VT/VF	28	Decreased scattering in Poincare plot before arrhythmic event	Rozen et al., 2013

PSVT, Paroxysmal supraventricular tachycardia; VF, Ventricular fibrillation; VT, Ventricular tachycardia; AF, atrial fibrillation; AVB, atrioventricular block; DFA, Detrended fluctuation analysis; pNN50, the number of pairs of successive beats that differ by more than 50 ms; SDNN, standard deviation of the average beating intervals; rMSSD, root mean square of successive differences; HF, high frequency; LF, low frequency; VLF, very low frequency.

* Beta coefficient is the slope between power spectra and VLF in log-log scale.

## Direct pharmacological inhibition of coupled-clock proteins of pacemaker cells causes changes in rate and rhythm

Direct pharmacological inhibition of coupled-clock proteins can induce arrhythmias. For example: (i) caging of intracellular Ca^2+^ by NP-EGTA in isolated rabbit pacemaker cells induces an increase in LCR variability and AP BI bradycardia together with arrhythmic events (Yaniv et al., 2011). (ii) A sudden increase in stochastic ryanodine receptor open probability, elicited by caffeine spritz in isolated rabbit pacemaker cells, induces tachycardia, together with arrhythmic events (Yaniv et al., 2013d). (iii) Specific PKA inhibitors (Younes et al., 2008) or CaMKII inhibitors (Yaniv et al., 2013c) superfused onto isolated rabbit pacemaker cells induce AP BI bradycardia together with arrhythmic events. (iv) Perturbing clock coupling in rabbit pacemaker cells by directly inhibiting either the M (ivabradine, an I_*f*_ inhibitor) or Ca^2+^ clock (cyclopiazonic acid, a SR Ca^2+^ pump inhibitor) produces increases in AP BI and AP BIV that are related to increases in LCR period and LCR period variability (Yaniv et al., 2014b). These results provide evidence that supports the coupled-clock theory, demonstrating the ability of the LCR Ca^2+^ signal to report the degree of synchronization between the two clocks, and how changes in the degree of synchronization lead to changes in AP BI and AP BIV.

## Reduced efficiency of intrinsic coupled-clock pacemaker mechanisms and arrhythmia

Similar to direct pharmacological inhibitors of coupled-clock proteins, mutation and genetically induced gene deletion of different components of the coupled-clock system are associated with arrhythmias *in vivo*.

### HCN gene

The hyperpolarization-activated channel (*I_f_*) consists of three HCN members (HCN1, HCN2, and HCN4) (Ludwig et al., 1998). HCN4 comprises the major fraction (70–80%) of SAN *I*_f_. Various mutations of human HCN4 channels are associated with arrhythmias, and with bradycardia in particular (Yeh et al., 2009; Schweizer et al., 2010; Duhme et al., 2013). Interestingly, the spontaneous cardiac beating rate of HCN4-knockout embryos is significantly slower than that of wild-type, but no arrhythmic events are observed. These results are in contrast to the conditional deletion of HCN4 in adult animals, where bradycardia is not evident but sinus pauses are detected (Stieber et al., 2004; Herrmann et al., 2007; Yeh et al., 2009) (Figure 1D). Other HCN transcripts that compose funny current channel in the mouse are HCN2, and a low level of HCN1. HCN2-deficient mice display mild cardiac dysrhythmia, both in the presence and absence of autonomic control of the heart rate (Stieber et al., 2004; Herrmann et al., 2007). Similarly, HCN1-deficient mice exhibit sinus dysrhythmia *in vivo* and in single isolated cells (Fenske et al., 2013). Interestingly, HCN1-deficient mice exhibit high beat-to-beat dispersion (quantified by Poincaré plots) that is typically observed in SAN dysfunction (Fenske et al., 2013). Therefore, HCN4 apparently is required to protect the heart from severe bradycardia and HCN2 and HCN1 are required to prevent arrhythmias.

Ivabradine is a specific *I*_*f*_ blocker that reduces the heart rate in patients, specifically patients with inappropriate sinus tachycardia to eliminate arrhythmia (Cappato et al., 2012). However, a recent study in isolated rabbit pacemaker cells demonstrated that ivabradine, even at a concentration that specifically inhibits *I_*f*_*, but does not directly suppress L-type current, SR Ca^2+^ cycling and other surface membrane ion channels, indirectly suppresses intracellular Ca^2+^ cycling (Yaniv et al., 2012, 2013b). The reduction in arrhythmic events is therefore likely due to drug effect on synchronization of functions within the coupled-clock system, and not simply to *I_f_*inhibition, *per se* (Yaniv and Lakatta, 2013).

### Ca_*v*_1.3

Voltage-gated Ca_*v*_1 channels (Ca_*v*_1.2 and Ca_*v*_1.3) mediate L-type Ca^2+^ channels that play distinct roles in mediating Ca^2+^ balance in the pacemaker cell. Both Ca_*v*_1.2 and Ca_*v*_1.3 channels are expressed in SAN, and Ca_*v*_1.3 expression in the atria and SAN cells is higher than in ventricular myocytes (Marger et al., 2011). Ca_*v*_1.3 current is activated faster and at more negative voltages than Ca_*v*_1.2 current and, therefore, in mice can contribute earlier during the diastolic depolarization (Christel et al., 2012). Bradycardia and arrhythmia are particularly prominent in Ca_*v*_1.3 knockout mouse pacemaker cells (Mangoni et al., 2003). Loss of function of Ca_*v*_1.3 both in mice and humans causes sick sinus syndrome (see above) and is characterized by severe bradycardia (Platzer et al., 2000; Baig et al., 2011). Moreover, patients with a mutation in the CACNA1D gene, which encodes the pore-forming α_1_ subunit of Ca_*v*_1.3, experience pronounced bradycardia in 12–24-h ECG recordings, and their HRV time-domain indices are increased (Baig et al., 2011) (Figure 1D). Note that functional significance of Ca_*v*_1.3 in large mammals has yet to be demonstrated.

### NCX1

The Na^+^/Ca^2+^ exchanger has two important roles in pacemaker cells: it not only maintains the cell Ca^2+^ balance by matching Ca^2+^ efflux to Ca^2+^ influx through the L-type Ca^2+^ current, but also contributes to diastolic depolarization (Maltsev et al., 2014). Specific knockout of SAN Na^+^/Ca^2+^ exchanger mice induces bradycardia and increases BIV in the proportion of mycoytes that express arrhythmic AP BI compared to control mice (Herrmann et al., 2013) (Figure 1D). Interestingly, numerical model simulations predict that only a reduction in Na^+^/Ca^2+^ exchanger density to below a specific threshold is accompanied by arrhythmic AP BI (Maltsev et al., 2013).

### TRPM4

TRPM4 is a monovalent nonselective cation channel permeable to Na^+^, K^+^, and Li^+^, but not to Ca^2+^ (Launay et al., 2002). Activation of TRPM4 channels that exist in murine pacemaker cells is achieved by both membrane depolarization and by a rise in intracellular Ca^2+^ (Hof et al., 2013). Although TRPM4 KO mice have heart rates similar to those of their controls, they exhibit a higher incidence of sinus pauses (Hof et al., 2013).

### Ryanodine channels

The stochasticity of spontaneous RyR activation determines the diastolic LCR Ca^2+^ signal and therefore the degree of synchronization of intracellular function of the coupled-clock system. A mutation in RyR, exon-3, in patients with catecholaminergic polymorphic ventricular tachycardia, is associated with arrhythmias (Bhuiyan et al., 2007) (Figure 1D). Isolated pacemaker cells from mice that express this mutation have a prolonged average AP BI with pauses between AP BIs together with an impaired chronotropic response to β adrenergic stimulation (Neco et al., 2012). Similarly, in inducible, heart tissue-specific RyR2 knockout mice, both *in vivo* ECG telemetry and *in vitro* isolated perfused heart, demonstrated bradycardic BI and arrhythmia (Bround et al., 2012).

### Shox2 and other transcription factors

The Shox2 transcriptional factor has been identified as a key regulator in pacemaker formation and differentiation (Liu et al., 2011). Shox2 gene KO mice have a significantly reduced heart beat rate and increased number of arrhythmic events (Liu et al., 2011) (Figure 1D). Moreover, deficient Shox2 transcription factor during development may cause abnormal of mouse SAN development associated with severe arrhythmias (Hoffmann et al., 2013). Therefore, the Shox2 gene also appears to be critical for normal pacemaking function. Other transcription factors than Shox2 are involved in pacemaker function. In this regard, expression of Tbx18 in guinea pig has been shown as an essential gene whose expression can convert quiescent cardiomyocytes to pacemaker cells (Kapoor et al., 2013), therefore, increasing the pacemaker-induced spontaneous beating rate of the cells and decreasing their BIV. Interestingly, Tbx18 transduction to the guinea pig embryonic cell lineage inhibits Cx43 expression, leading to significant electrical uncoupling (Kapoor et al., 2011).

### Ankyrin-B

Ankyrins are adaptor proteins that are required for targeting channels and transporters in pacemaker cells to the membranes in which they function. Human patients with ankyrin-B-deficiency have highly penetrant sinus node dysfunction coupled with increased susceptibility to spontaneous and inducible atrial fibrillation (Le Scouarnec et al., 2008). Interestingly, ankyrin-B -deficient mice also have reduced expression of Na^+^/Ca^2+^ exchanger and Na^+^/K^+^ ATPase (Le Scouarnec et al., 2008). Finally, cells isolated from ankyrin-B-deficient mice have increased BIV (Cunha et al., 2011) (Figure 1D). Thus, down regulation of ankyrin-B induces abnormal membrane organization that is implicated in a reduced efficiency of pacemaker clock coupling that causes abnormal electrical activity within SAN.

### Cell-to-cell uncoupling mechanisms

As described above, the BIs of pacemaker cells residing in the SAN become entrained by electrotonic and mechanical cell-to-cell interactions within the tissue (Jalife, 1984; Watanabe et al., 1995). Numerical model simulations predict that cardiac arrhythmias can occur when normal coupling between pacemaker cells in SAN tissue is perturbed (Ostborn et al., 2001). Cardiac diseases, and specifically sick sinus syndrome, are associated with reduction in cell-cell junctional proteins (Dobrzynski et al., 2007). ECG of mice with a cardiac conduction-specific knockout of desmoplakin, a protein that affects mechanical cell-to-cell interaction in the cardiac conduction system, exhibits sinus arrhythmias characterized by a strikingly increased number of sinus pauses compared to wild-type mice (Sheikh et al., 2013).

### Passive mechanisms

Although connexin43 is absent in the center of the pacemaker tissue, it is expressed in the peripheral area. A reduction in connexin 43 in aged guinea pig SAN is associated with a reduction in heart rate and an increase in arrhythmogenic events (Jones et al., 2004). Moreover, pacemaker tissue contains functional gap junctions and connecting cardiac fibroblasts (Camelliti et al., 2004). Because an increase in fibroblasts expression can slow the generation of pacemaker excitability, it may be involved in bradycardia and sick sinus syndrome (for review see Kohl et al., 2005). A detailed review of the role of passive pacemaker tissue properties on its electrical conductance is present in this issue (Unudurthi et al., 2014).

## Reduced efficiency of synchronization of activity across populations of cells and arrhythmia

High-resolution optical mapping of SAN tissue has helped to resolve how reduced synchronization of activity across populations of cells within the SAN can induce arrhythmia. In this regard, different intrinsic mechanisms can be involved in tachy-brady heart-rate alteration and exit block that leads to long sinus pauses and increases susceptibility to cardiac arrhythmias: (i) an increase in adenosine level in human SAN tissue, an endogenous metabolite of the heart, through adenosine A1 receptor upregulating, can lead to SAN dysfunction (Lou et al., 2013, 2014); (ii) an increase in B-type and C-type natriuretic peptides increase the mice SAN conduction velocity and shift the initial exit site (Azer et al., 2014). (iii) Mutation in Ca^2+^-binding protein calsequestrin 2 is associated with different cardiac diseases. In calsequestrin knockout mice the SAN exhibits bradycardia, conduction abnormalities and increase beat-to-beat variability (Glukhov et al., 2013). (iv) Ganglion nerve plexi can stimulate the intrinsic cardiac nervous system. In mice pulmonary vein ganglia stimulation shifts the origin of SAN discharges and decreases the heart rate (Zarzoso et al., 2013). (v) Impaired SR function in canine failing hearts results in an impaired shift in the location of the pacemaker site in response to β-AR stimulation (Shinohara et al., 2010).

## Summary

Changes in heart rate and rhythm are harbingers of the appearance of arrhythmogenic events. Reduction in the degree of synchronization of any intrinsic clock functions of pacemaker cells or in the synchronization among pacemaker cells residing in the SAN can be associated with arrhythmia occurrence. The extent to which restoring normal synchronization of intrinsic clock periods within pacemaker cells and among pacemaker cells can prevent arrhythmogenic events awaits further elucidation. In our opinion, future work requires a focus on the connection between dysfunction of inherent intrinsic mechanisms associated with different cardiac diseases and cardiac arrhythmias. This connection can be explored in genetically manipulated mouse models, in animals like rabbit, dog and sheep with heart failure or atrial fibrillation, and in human-derived cardiomyocytes or human SAN. This knowledge will contribute greatly to our understanding of cardiac impulse initiation in health and in cardiac disease.

### Conflict of interest statement

The authors declare that the research was conducted in the absence of any commercial or financial relationships that could be construed as a potential conflict of interest.
